# Necessity of Cerebrospinal Fluid Analysis in Young Children Aged 6-60 Months Presenting With Febrile Convulsions: A Systematic Review

**DOI:** 10.7759/cureus.89861

**Published:** 2025-08-12

**Authors:** Ramesh Patidar, Madhuri Shende, Saroj Rajaram, Prashant R Kadam, Shreeya P, Reena Rajput

**Affiliations:** 1 Pediatrics and Child Health, Zulekha Hospital, Sharjah, ARE; 2 Pediatrics and Child Health, Khurshitji Beharamji (KB) Bhabha Hospital, Mumbai, IND; 3 Critical Care, New Medical Centre (NMC), Dubai, ARE; 4 Pediatrics and Child Health, Bombay Hospital Institute of Medical Sciences (BHIMS), Mumbai, IND; 5 School of Medicine, University of Central Lancashire, Preston, GBR; 6 Pediatrics, Royal College of Paediatrics and Child Health, London, GBR

**Keywords:** cerebrospinal fluid analysis, febrile convulsion, febrile seizures, lumbar puncture, pediatric neurology

## Abstract

Febrile seizures (FS) are common in children aged 6-60 months and often cause concern because of their potential association with central nervous system (CNS) infections like occult meningitis. While most FS are benign, distinguishing simple from complex cases is significant.

This systematic review aims to evaluate the diagnostic utility and necessity of cerebrospinal fluid (CSF) analysis in young children presenting with FS to guide appropriate clinical decision-making.

This systematic review followed the Preferred Reporting Items for Systematic Reviews and Meta-Analyses (PRISMA) guidelines where literature was searched in PubMed, Cochrane Library, Web of Science, and Google Scholar (2015-2025) using terms related to FS, CSF analysis, and children aged 6-60 months. Inclusion criteria focused on observational studies that reported lumbar puncture. Two reviewers independently screened studies and extracted data on seizure type, age, and CSF results.

Over 1,306 records were screened, and nine studies were included that involved 3,143 children aged 6-60 months. CSF analysis usage varied widely from over 6% to 100%. Bacterial meningitis was detected in 0.7-14% without clinical signs. High yield was among infants under 18 months. The risk of bias was low in most studies.

CSF analysis in FS should be guided by age, vaccination status, and clinical signs. A selective approach avoids unnecessary lumbar punctures while ensuring serious CNS infections are not missed.

## Introduction and background

Febrile seizures (FS) are the most prevalent type of seizure disorder in childhood, commonly happening between the ages of six and 60 months. They are characterized by convulsions related to fever in the absence of intracranial infections, metabolic disturbances, or an unprovoked seizure history [1]. FS is a major concern for parents, as, in most cases, they may be benign and self-limiting. FS affects over 2-5% of children aged between six and 60 months globally [1]. These seizures are common in males, as the male-to-female ratio was about 1.2:1 [2]. The FS incidence is high in children under two years of age, between 12 and 18 months [3]. Febrile illnesses, particularly viral infections like upper respiratory tract infections and gastroenteritis, are the most common triggers [2,4]. A family history of FS is also a known risk factor that has indicated a possible genetic predisposition.

FS are classified into two categories: simple febrile seizures (SFS) and complex febrile seizures (CFS). SFS are tonic-clonic seizures that last less than 15 minutes and happen once a day for about 80-85% of FS cases [5]. Additionally, CFS can be characterized by focal features, a duration of more than 15 minutes, or recurrence within 24 hours [1]. Differentiating between these categories is essential as CFS might need more neurological evaluation and imaging. High rates of incidence are observed in children under 20 months, and FS affects children between the ages of six and 60 months [2,3]. This age-specific vulnerability may be the result of the immature brain's heightened sensitivity to fever-induced neuronal excitability. The predominance of bacterial meningitis in most countries has been significantly decreased by the extensive use of vaccines, especially against *Haemophilus influenzae* type b and *Streptococcus pneumoniae*. This pattern has influenced the clinical decision-making process around performing lumbar puncture (LP) in children presenting with FS.

Central nervous system (CNS) infections pose diagnostic challenges because of their different clinical presentations as well as the need for rapid, accurate identification to guide treatment. Cerebrospinal fluid (CSF) analysis is critical in diagnosing these infections, particularly in the detection of occult meningitis. Occult meningitis is a form of meningitis without outward symptoms, which poses a diagnostic challenge. Pleocytosis refers to an increased white blood cell count in the CSF, which may indicate inflammation. Polymerase chain reaction (PCR) is a molecular diagnostic method used to detect small amounts of genetic material from pathogens; while highly sensitive, it can occasionally yield false positives [6]. The diagnostic importance of CSF analysis was its ability to differentiate causative pathogens, which is significant for timely and appropriate therapeutic decisions.

Examination of CSF includes the assessment of white blood cell count, protein concentration, and glucose and lactate levels which helps in distinguishing bacterial, viral, fungal, or other infectious etiologies [7]. Direct microscopic examination and CSF culture helped in the identification of specific organisms and the determination of their antibiotic sensitivities as this provided targeted treatment methods [6,7]. Additionally, advanced diagnostic techniques like nested PCR assays helped in detecting multiple pathogens simultaneously. However, these molecular methods are sensitive as they have the risk of false-positive results mainly in bacterial meningitis that may complicate clinical interpretations [8]. Identification of occult meningitis was one of the clinical challenges. Traditional CSF markers lack specificity. This makes it difficult to diagnose meningitis in the absence of classical symptoms [6]. Identifying occult meningitis remains challenging due to the low specificity of traditional CSF markers and absence of classical symptoms [6]. Due to this, there is a shift to sensitive PCR-based diagnostic methods. This can lead to misdiagnosis and inappropriate usage of antimicrobials [8]. Additionally, current CSF biomarkers for neuroinflammatory conditions like those related to HIV-related neurocognitive disorders are still under investigation and not yet combined into routine clinical practice [6]. These limitations state the necessity for improved diagnostic biomarkers and standardized protocols to improve the accuracy of CSF analysis in CNS infections. CSF analysis is indispensable for diagnosing CNS infections, mainly occult meningitis. Ongoing research for refining the diagnostic tools and validating the novel biomarkers is significant for better clinical outcomes [6,8]. This review evaluated the diagnostic yield and necessity of performing LP for CSF analysis in children aged between six and 60 months who present with FS.

## Review

Methodology

Study Design

This study was designed and conducted as a systematic review by following the Preferred Reporting Items for Systematic Reviews and Meta-Analyses (PRISMA) guidelines. 

Search Strategy

A literature search was performed across different electronic databases including PubMed, Cochrane Library, Web of Science, and Google Scholar, from 2015 to May 2025. The search strategy combined Medical Subject Headings (MeSH) terms and free-text keywords related to FS, CSF analysis, LP, and the target age group.

Key search terms included febrile seizure OR febrile convulsion OR fever seizure AND cerebrospinal fluid OR CSF OR lumbar puncture OR spinal tap AND infant OR child OR pediatric OR paediatric. Search filters were applied to limit results to human studies. Reference lists of included studies and related systematic reviews were manually screened for more eligible studies.

This review was not registered in the International Prospective Register of Systematic Reviews (PROSPERO) due to the retrospective design but followed PRISMA 2020 guidelines. The PRISMA checklist and the full search strings for each database, along with filters for language and study type, are provided in Appendix A. The excluded studies with reasons are included in Appendix B. 

Study Selection Criteria

This systematic review included studies involving children aged 6-60 months who presented with FS, classified as either simple or complex. FS was defined as seizure episodes in relationship with fever (≥38°C or 100.4°F), without a prior history of afebrile seizures, and in the absence of acute CNS infections or underlying neurologic abnormalities.

Inclusion criteria: This review included the study population where children aged 6-60 months presented with FS, irrespective of seizure type (simple or complex) and whether it was a first or recurrent episode. The study design includes observational studies, including prospective or retrospective cohort studies, cross-sectional studies, and medical chart reviews. The assessment or intervention in this review included studies that performed LP or CSF analysis as part of clinical evaluation. The studies that reported the diagnostic yield of CSF analysis, such as the detection of bacterial or viral meningitis, CSF pleocytosis, or other relevant CSF abnormalities, were also included in this study. Only articles published in English were considered in this review.

Exclusion criteria: Studies that did not specify the age range of participants or included children outside the 6-60-month window were excluded. Studies that involved children with pre-existing neurologic disorders that were diagnosed before FS were not included. Studies including children with known immunosuppression, malignancy, or immunodeficiency syndromes were excluded. Studies with patients with a pre-diagnosed CNS infection, such as bacterial meningitis or viral encephalitis, before LP were also excluded. Case reports, opinions, narrative reviews, conference abstracts without full data, or editorials were not included in this review. Animal studies or studies lacking sufficient data on CSF analysis outcomes were also not included.

Study Selection

Titles and abstracts were examined by two reviewers to ensure they met the predetermined qualifying requirements. We retrieved and examined full-text articles of studies which may be eligible. Another examiner or conversation was used to resolve disagreements. The study selection procedure is shown in the PRISMA flow diagram.

Data Extraction

Data extraction was made using a standardized extraction form in MS Excel (Microsoft Corp., Redmond, WA, USA). For each study, data were extracted on study characteristics such as author, year of publication, country of study, study design, sample size, age range, seizure type distributions, and CSF analysis performed, and the key findings of each article were also noted. Subgroup analyses were also extracted, including age-based outcomes, seizure type distributions, and correlations between neurologic signs and CSF findings. Subgroup analysis was also extracted with subgroup criteria, number of studies, CSF analysis yield, and clinical implication.

Results

**Figure 1:**
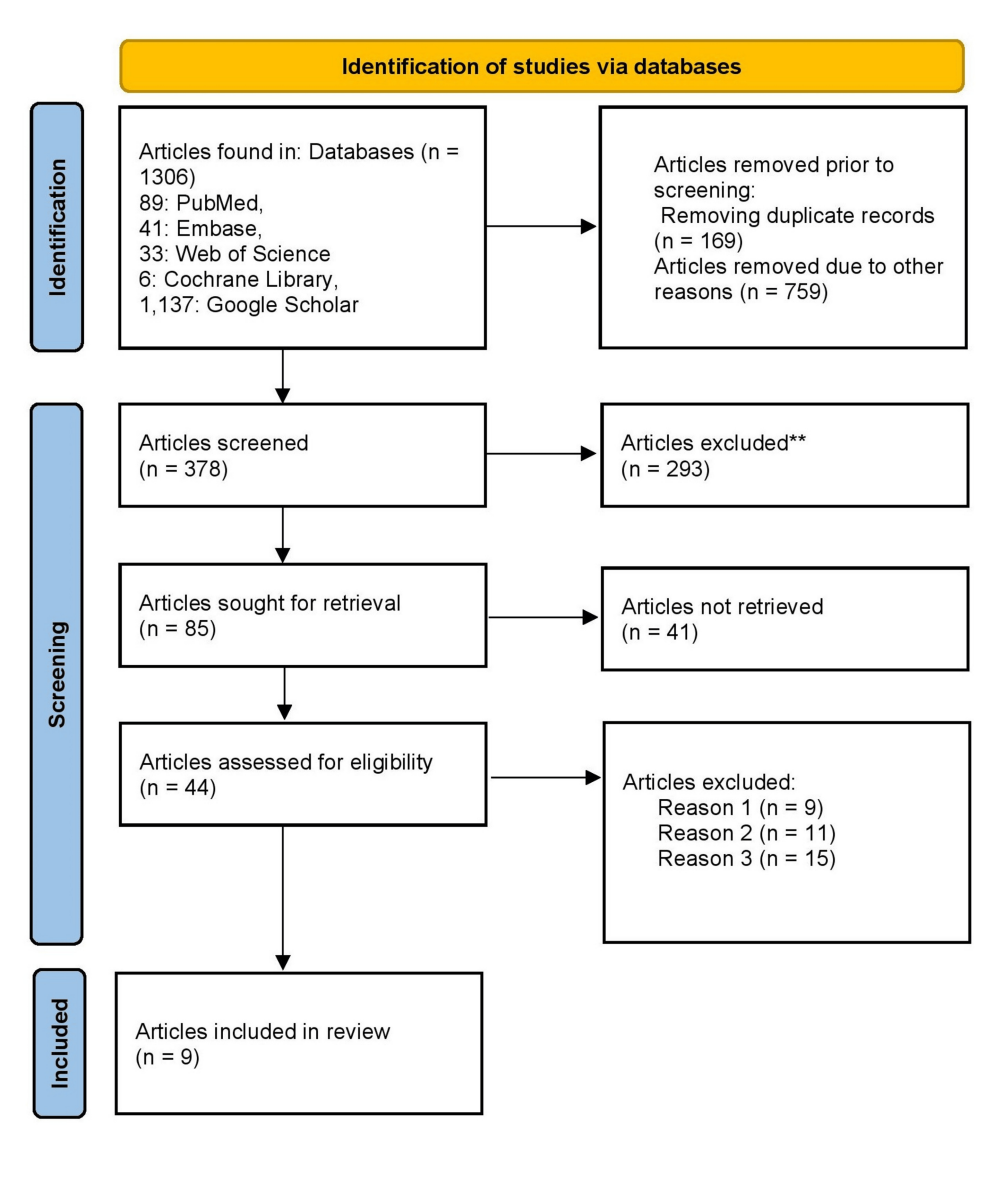
PRISMA flowchart **Articles not meeting the eligibility criteria, which included factors like irrelevance to the study topic or inadequate reporting. PRISMA: Preferred Reporting Items for Systematic Reviews and Meta-Analyses

Over 1,306 records were observed using the database searches. Before the screening process, 169 duplicate records were removed, along with an additional 759 records excluded for other reasons such as incomplete data, irrelevant document types, or language restrictions. There were 378 records that were screened based on titles and abstracts. In that, 293 records were excluded for not meeting the eligibility criteria, which included factors like irrelevance to the study topic or inadequate reporting. The 85 articles were sought for full-text retrieval; however, 41 could not be retrieved due to issues such as limited access or missing files. Thus, 44 full-text articles were assessed for eligibility. Out of these, 35 articles were excluded for the following reasons: nine for not meeting inclusion criteria, 11 for inappropriate population or outcomes, and 15 for unsuitable study design. Finally, nine studies were included in the systematic review. Figure 1 presents the PRISMA 2020 flowchart detailing the study selection process.

A total of 3,143 children aged 6-60 months who presented with FS were observed in the included articles. The studies were observed in regions such as India, France, South Korea, Pakistan, and Australia. Six studies were retrospective in design, while three studies used prospective methodologies. The prevalence of CSF analysis differed across the studies, ranging from 6% to 100% of included patients. Table 1 presents the characteristics of the included studies and the characteristics of the patients.

**Table 1:** Characteristics of the included studies CSF: cerebrospinal fluid; CFS: complex febrile seizure; FS: febrile seizures; SFS: simple febrile seizure; LP: lumbar puncture; ABM: acute bacterial meningitis; CNS: central nervous system; HSV-ME: herpes simplex virus meningoencephalitis; EEG: electroencephalogram; URTI: upper respiratory tract infection

Study	Study type	Country	Sample size	Age range (months)	Seizure type distribution	CSF analysis performed	Key findings
Patadia et al., 2021 [9]	Cross-sectional	India	50	6-18	40 simple (80%), 10 complex (20%)	Yes	14% (7/50) had ABM. 3/7 ABM cases had meningeal signs. Only 1 culture positive (*Klebsiella*). The ABM group had significantly higher temperature, heart rate, and respiratory rate compared to the non-ABM group (p<0.05)
Stephen et al., 2015 [10]	Prospective observational	India	120	6-18	Simple (64.2%), atypical (35.8%)	Yes (all patients)	ABM: 4.2% (n=5). Probable CNS infection (non-bacterial): 5% (n=6). None had clinical signs of meningitis
Guedj et al., 2017 [11]	Multicenter retrospective cohort	France	839	6-60	CFS: focal, prolonged ≥15 minutes, or multiple in 24 hours	260/839 (31%) underwent LP	Bacterial meningitis: 0.7% (5/715); HSV-ME: 0% (0/657). All 5 meningitis cases had prolonged seizures as a component
Kim et al., 2024 [12]	Retrospective observational	South Korea	253	6-60	SFS (73.9%), CFS (26.1%)	Yes (all 253 cases)	3.9% had CSF pleocytosis. No cases of bacterial meningitis. 7 cases (2.8%) of enteroviral meningitis; only 1 had pleocytosis. No significant difference in CSF pleocytosis across age groups. EEG abnormalities were minimal and not clinically significant
Mustafa et al., 2023 [13]	Cross-sectional	Pakistan	156	6-18	The first episode of SFS	Yes, LP with CSF analysis for all participants	Meningitis was found in 16 (10.3%) children
Son et al., 2018 [14]	Retrospective chart review	South Korea	1249	Mean 12.05±9.13; 66 <12 months	SFS	Yes (in 75 patients)	66/75 (88%) who had LP were <12 months. 5/75 (6.7%) showed CSF pleocytosis. 3 (4%) had bacterial meningitis (ages 4, 8, 12 months) despite being immunized and without neurologic signs
Suryavanshi et al., 2020 [15]	Prospective hospital-based	India	120	6-60	Generalized tonic-clonic (86), focal (22), atonic (2)	Yes	69.1% were male. Most cases were aged 6-12 months. URTI (45%) was the most common cause. Meningitis was diagnosed in 23 (19.2%) cases
Guedj et al., 2015 [16]	Retrospective, cross-sectional, observational	France	205	6-11	First SFS	Yes (61 patients; 29.8%)	No bacterial meningitis detected; CSF pleocytosis in 18%; no bacterial growth
Francis et al., 2016 [17]	Observational study	Australia	151	6-57	Simple (65%), complex (33%)	Yes (9/151; 6%)	Virus detected in 71% (rhinovirus, adenovirus, and enterovirus were the most common). Viral coinfections in 34%. FS occurred in 11% within 14 days post-immunization. No pathogen-specific clinical differences noted

Patadia et al. conducted a cross-sectional study of 50 children aged 6-18 months in India and observed that 14% had acute bacterial meningitis; furthermore, only three of these seven cases presented with clinical meningeal signs. One case showed positive bacterial culture (*Klebsiella*), and this states the diagnostic challenges in this population. The CSF analysis was significant in the first FS between six and 18 months [9].

Stephen et al. performed a prospective observational study of 120 children aged 6-18 months and observed acute bacterial meningitis in 4.2% and CNS infection in 5% of the cases. However, none of the patients with confirmed infections had clinical signs of meningitis at presentation. This states the occult nature of serious CNS infections in this age group. The study showed no significant difference in infection rates between SFS and atypical presentations. This suggests that seizure type alone should not determine the need for CSF analysis [10]. The study by Guedj et al. analyzed 839 children aged 6-60 months with CFS across France. The study identified bacterial meningitis in only 0.7% of cases, with all five cases in patients with long seizures as a component. No cases of herpes simplex virus (HSV) encephalitis were observed among the 657 tested patients [11]. Kim et al. conducted a retrospective analysis of 253 South Korean children aged 6-60 months undergoing CSF analysis. The study showed CSF pleocytosis in 3.9% of cases. However, enteroviral meningitis was detected in 2.8% of patients; additionally, only one case showed pleocytosis. The study identified no significant differences in CSF abnormalities across different age groups [12].

Mustafa et al. evaluated 156 Pakistani children aged 6-18 months presenting with first-episode SFS and performed CSF analysis on all participants. The study showed a high meningitis rate of 10.3%. This suggests regional variations in infection prevalence and supports an approach to CSF analysis [13]. Son et al. observed SFS by analyzing 75 patients who underwent LP from 816 cases. The study showed CSF pleocytosis in 6.7% and confirmed bacterial meningitis in 4% of tested patients. All three meningitis cases happened in children aged four, eight, and 12 months who were fully immunized and lacked neurological signs. This emphasizes the diagnostic challenge in well-appearing infants [14]. Suryavanshi et al. studied 120 children aged 6-60 months with various seizure types. The results showed abnormal CSF consistent with meningitis in 19.2% of cases. The study observed the highest yield in children under 12 months and recommended routine CSF analysis for all atypical presentations. Upper respiratory tract infections were identified as the most common precipitating factor followed by other febrile illnesses [15]. Guedj et al. analyzed 205 French infants aged 6-11 months with first SFS, performing LP in 29.8% of cases. No bacterial meningitis was detected, and CSF pleocytosis occurred in only 18% without bacterial growth, leading the authors to conclude that routine LP is not necessary in this specific population [16]. Conversely, Francis et al. studied 151 Australian children aged between six months and five years, performing CSF analysis in only 6% of cases with negative results in all tested patients. This study emphasized the predominant role of viral infections in FS, particularly rhinovirus, adenovirus, and enterovirus, while noting that FS occurred in 11% of children within 14 days post-immunization [17]. Table 2 presents a subgroup analysis highlighting indications for CSF analysis.

**Table 2:** Subgroup analysis: indications for CSF analysis in children with FS SFS: simple febrile seizure; AFS: atypical febrile seizure; ABM: acute bacterial meningitis; CNS: central nervous system; CSF: cerebrospinal fluid; HSV-ME: herpes simplex virus meningoencephalitis; WBC: white blood cell count; LP: lumbar puncture; FS: febrile seizures

Study	Subgroup criteria	Number of studies	CSF analysis yield	Clinical implication
Patadia et al., 2021 [9]	Type of seizure (simple vs. complex); presence of meningeal signs; age group (6-12 vs. 13-18 months)	Single center	14% (7/50)	CSF analysis is critical in the first FS between 6 and 18 months to rule out ABM, even in the absence of meningeal signs
Stephen et al., 2015 [10]	Age 6-12 months: Children aged 6-12 months	Single center	ABM: 4.3%; probable CNS: 5.4%	Consider CSF even without clinical signs; bacterial meningitis may be occult. Lower incidence, but CSF still advisable in atypical presentations. Even simple FS can have CNS infection without meningeal signs. Slightly higher infection rate; CSF strongly recommended
	Age 13-18 months: Children aged 13-18 months		ABM: 3.6%; probable CNS: 3.6%	
	SFS: Generalized, <15 minutes, no recurrence		ABM: 3.9%; probable CNS: 3.9%	
	AFS: Prolonged, focal, or recurrent		ABM: 4.7%; probable CNS: 7%	
Guedj et al., 2017 [11]	All CFS cases	Single center	Bacterial meningitis: 0.7%; HSV-ME: 0%	Low diagnostic yield overall for serious CNS infections. Extremely low risk when exam is not suggestive of CNS infection. Isolated multiple seizures not associated with meningitis. Higher LP rate when physical exam raised concern. LP often deferred in the absence of concerning clinical signs. Prolonged seizures are a key risk factor; consider LP, especially in this subgroup. Increased vulnerability in younger children; LP should be considered
	Normal clinical exam		Bacterial meningitis: 0%; HSV-ME: 0%	
	Multiple seizures only		Bacterial meningitis: 0%; HSV-ME: 0%	
	With clinical signs		LP performed in 113 (54%)	
	Without clinical signs		LP performed in 147 (23%)	
	Prolonged seizure component		All 5 bacterial meningitis cases occurred in this group	
Kim et al., 2024 [12]	Age groups: 6-12 months, 13-18 months, 19-60 months	Single center	Pleocytosis: 10/253 (3.9%)	LP should not be routine in all first FS cases, even under 12 months. Consider selectively based on clinical suspicion (e.g., altered mental status, neurologic signs), not just age or CFS type
	Age- and seizure-based		Enterovirus detected: 7/253 (2.8%)	
	Age at first FS			
	Type (SFS/CFS)			
			Bacterial meningitis: 0/253	
	Clinical signs (consciousness, neuro exam)			
Mustafa et al., 2023 [13]	Stratified by age group (6-12 months vs. 13-18 months), gender, weight, etc.	Single center	10.3% positive for meningitis	A high index of suspicion warranted; 1 in 10 children had meningitis
Son et al., 2018 [14]	Children <12 months with SFS who underwent LP	Single center	5/75 (6.7%) showed pleocytosis; 3/75 (4%) confirmed bacterial meningitis	LP should be considered in all SFS cases <12 months, even if immunizations are up-to-date and no neurologic signs are present
Suryavanshi et al., 2020 [15]	Age: 6-12 months (63), 12-24 months (32), >24 months (25)	Single center	Raised WBC in 29/120 cases (24.2%). CSF abnormal in 23 (19.2%), consistent with meningitis	Routine CSF analysis helps rule out CNS infections in CFS; high yield in <12 months; consider in all atypical cases
	CFS: Seizure >15 min, focal, or recurrent in 24 hours			
	CSF cells >5/mm³, protein >40 mg%, sugar <2/3 blood glucose for meningitis			
Guedj et al., 2015 [16]	Infants 6-11 months with first SFS, no signs of meningitis	Single center	29.8	Routine LP is not routinely necessary in this age group with the first SFS; the risk of bacterial meningitis is extremely low
Francis et al., 2016 [17]	Children aged 6 months to 5 years with FS	Single center	Negative in all cases	Viral infections are common in FS, but CSF infection is rare; FS is infrequently related to recent vaccination

Risk of Bias

**Figure 2:**
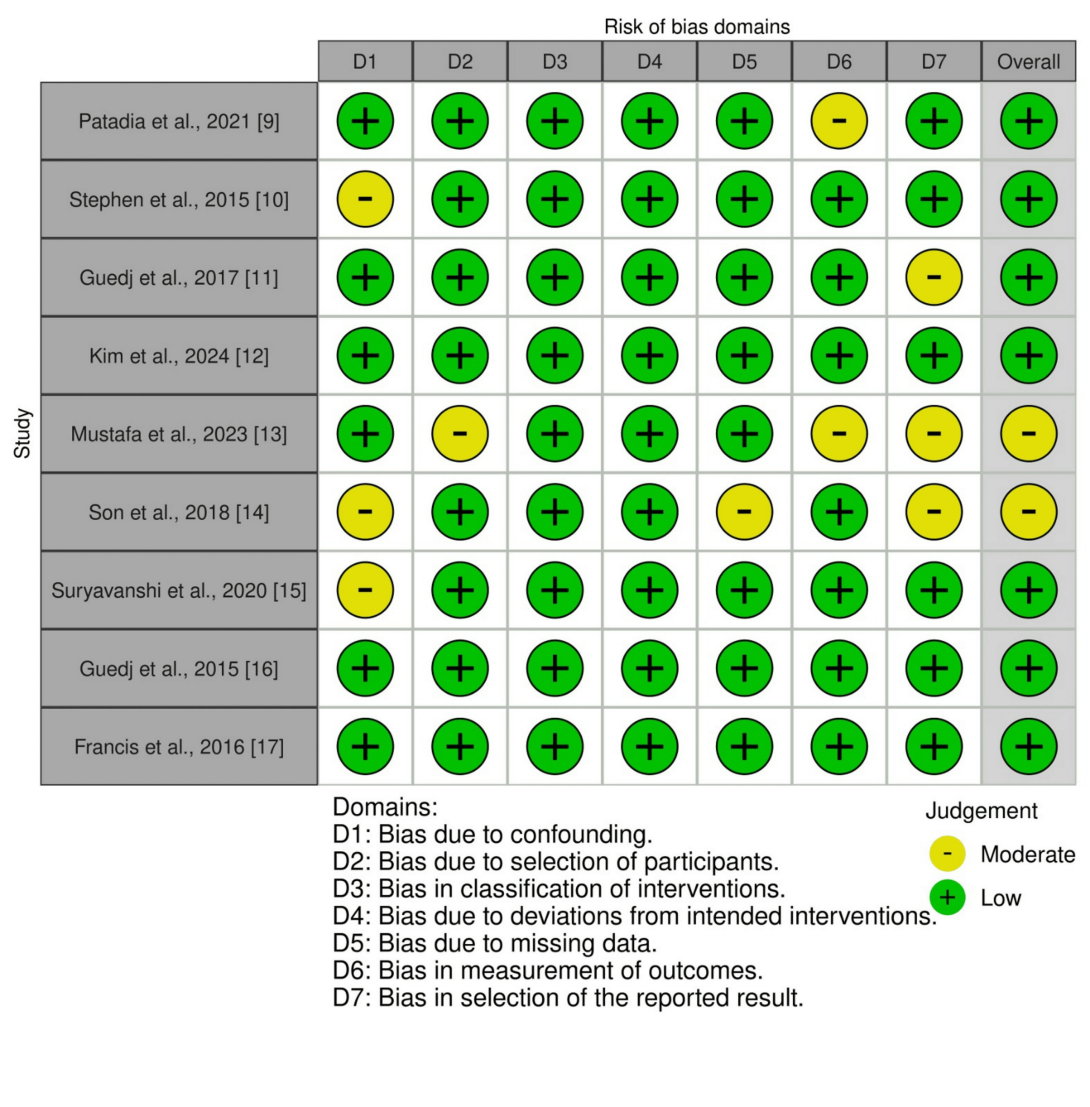
Risk of bias of the studies included

**Figure 3:**
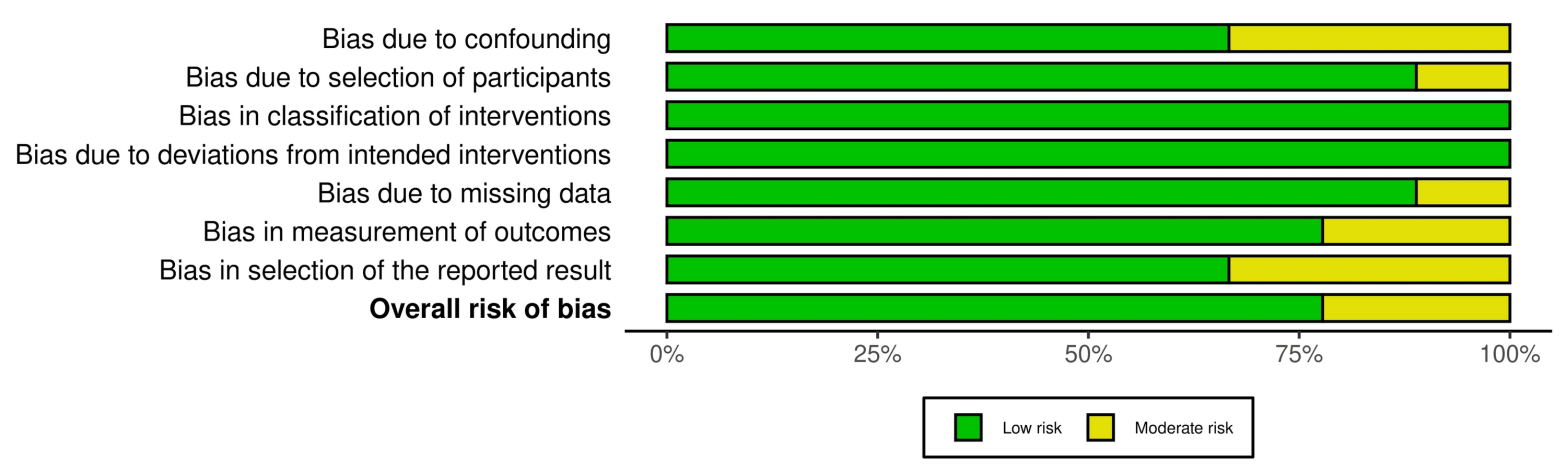
Risk of bias of the included studies using ROBINS-I ROBINS-I: Risk Of Bias In Non-randomized Studies-of Interventions

The risk of bias was evaluated for the included studies using the Risk Of Bias In Non-randomized Studies-of Interventions (ROBINS-I). Here, over five studies (55.5%) have a low risk of bias [9-11,15,16]. Two studies (22.2%) by Mustafa et al. and Son et al. evaluated a moderate overall risk of bias because of concerns in specific domains such as confounding and selection of reported results [13,14]. The study by Guedj et al. showed moderate risk in domain D7 but was still rated overall as low risk [11]. All studies showed low bias including intervention classification, intended intervention deviations, and missing data. The most common areas of concern were confounding and selection of the reported result, which slightly impacted the overall judgment in some cases. Detailed risk of bias assessments are provided in Figure 2 and Figure 3.

Discussion

This systematic review provides important information regarding the utilization of diagnostics and the necessity of CSF analysis in children aged 6-60 months presenting with FS. The results showed some variability in the prevalence of CNS infections across different populations and healthcare settings. This underscores the complexity involved in creating guidelines for performing LP in this vulnerable population.

The prevalence of bacterial meningitis in children with FS varied significantly across the included studies, from 0% to 14%. The high rates were observed in developing countries, as Patadia et al. observed that over 14% of bacterial meningitis was reported [9]. Similarly, Mustafa et al. in Pakistan have reported 10.3% meningitis [13]. These findings are observed to be opposite to studies carried out in developed countries such as Guedj et al. in France [16]. Here, no cases of bacterial meningitis were observed among infants aged between six and 11 months with SFS.

The geographical difference in the prevalence of infection is similar to the global epidemiological trends, and this may show the differences in vaccination, access to healthcare, and the baseline burden of infectious disease [18]. The usage of vaccines against *Haemophilus influenzae* type b and *Streptococcus pneumoniae *has decreased bacterial meningitis in developed countries [19]. However, the persistence of a highly resource-limited environment states the significance of regional epidemiological factors when developing clinical guidelines.

The present review observes that the risk of serious CNS infections differs with age within the 6-60-month period. The studies like Son et al. and Suryavanshi et al. show high rates of infections under 12 months [14,15]. This is similar to the study by Smith et al. which observed that children under 12 months have a high risk for occult bacterial meningitis in terms of FS [20]. The age-related vulnerability may be because of factors like immature responses of the immune system, incomplete vaccination schedules, and the blood-brain barrier's increasing permeability in young children [21]. Research by Capovilla et al. also emphasized that the neurological immaturity in infants under 12 months makes a situation where the signs of traditional meningeal may be absent even in the presence of serious infections of the CNS [22]. Children under 12 months or those with incomplete immunization, particularly against *Haemophilus influenzae* type b and *Streptococcus pneumoniae*, are at higher risk for bacterial meningitis. In such cases, CSF analysis is crucial to rule out serious underlying infections.

One of the significances of this review is the high prevalence of occult bacterial meningitis infections without common clinical symptoms of meningitis. Stephen et al. and Son et al. showed that children with bacterial meningitis presented without any neurological symptoms or meningeal irritations [10,14]. This is similar to the study by Sawires et al. and Muttath et al. on the unreliability of clinical examination without considering the CNS infections in young children with FS [23,24]. The limitations of occult meningitis have been stated in emergency department studies, where the clinical decision rules are based on the physical examination. This showed poor sensitivity for the detection of bacterial meningitis in children with FS [25,26]. This states the limitations of clinical assessments and also supports the consideration of diagnostic approaches, especially in high-risk populations.

The association between the types of seizure and CNS infection remains an issue. Some studies have also suggested that the high levels of infections in the CFS have not identified any significant differences between the simple and complex presentations. This contradicts the previous recommendations that strongly emphasized seizure complexity as the major justification for LP [27]. The study by Jarrett et al. observed no significant differences in rates of bacterial meningitis between children with SFS and CFS when controlling for age and clinical presentation [28]. Similarly, the Consequences of Prolonged Febrile Seizures in Childhood (FEBSTAT) study suggested that fever-related epilepticus rarely causes significant CSF pleocytosis. Also, the use of seizure duration alone is a primary decision criterion [29]. A recent systematic review by Corsello et al. in Pediatric Neurology emphasized similar findings regarding the limited predictive value of CFS for CNS infections and called for updated LP criteria that consider regional vaccination coverage and newer diagnostic technologies [18].

Significant differences in infection rates were observed in different countries and healthcare systems that state the importance of region-specific guidelines. Studies from sub-Saharan Africa and South Asia observed increased rates of bacterial meningitis in children with FS when compared to developed countries [30]. These differences highlighted the variations in vaccination coverage, nutritional status, and access to healthcare.

This systematic review includes studies from various regions and healthcare settings, improving the generalizability of studies on CNS infections in children with FS. Many studies used large samples [14,28], thus improving the validity of the result. Standardized diagnostic criteria were used in studies like Mahajan et al. and Powell et al. that ensured the reliability [25,26]. Research from both high-resource [16] and low-resource settings [13] provided valuable contrasts. Stratification by age and seizure type helped in a better understanding of risk that supports age- and region-specific clinical guidelines.

Some limitations have been observed. The studies that were included in this review differed in design, characteristics of the population, and diagnostic methods. This limits the ability to perform meta-analysis or to derive definitive conclusions. The retrospective nature of most included studies shows some selection bias and the difference in the rates of LP. This suggests that the clinical decision-making was not standardized. Even with these limitations, various clinical implications can be observed from this review. First, the bacterial meningitis risk in children with FS differs based on age, geographic location, and healthcare environment. Second, the clinical examination alone is not sufficient for ruling out CNS infections, especially in children under 12 months. Furthermore, the current guidelines need to be updated to show the regional epidemiological differences and advancements in diagnostic technology.

There is a need for future research for optimizing the management of children with FS. Large-scale prospective studies are necessary for defining the risk factors for CNS infections in various populations and healthcare environments. The development and validation of clinical decision guidelines that include several risk factors other than age and seizure type could increase diagnostic accuracy while preventing unnecessary treatments. Novel diagnostic approaches include methods such as point-of-care C-reactive protein (CRP) testing, procalcitonin assays, multiplex PCR panels, and advanced neuroimaging techniques like diffusion-weighted MRI and CT perfusion scans. These methods enable rapid, bedside evaluation and the detection of CNS infections. In selected cases, they may reduce the need for immediate LP. This may give an alternative to traditional LP in some selected cases. Furthermore, studies examining the long-term outcomes of children with FS, including those who did and did not undergo LP, are essential to inform risk-benefit analyses and guide clinical practice recommendations. Despite technological advances, current CSF biomarkers lack specificity and sensitivity in the early detection of bacterial meningitis, especially in asymptomatic cases. There is a growing need for rapid, bedside-compatible diagnostic tools and validated decision-making algorithms.

## Conclusions

This systematic review states the role of CSF analysis in evaluating FS among children aged 6-60 months. While most FS, particularly simple ones, are benign and self-limiting, certain subgroups, especially infants under 18 months, have presented a diagnostic dilemma because of the occult nature of CNS infections. The included studies show that bacterial or viral meningitis may occur without classical signs, which include neck stiffness, photophobia, bulging fontanelle, and positive Kernig's or Brudzinski's sign, highlighting that seizure type alone is insufficient for ruling out serious CNS pathology. However, the overall diagnostic yield of routine LP in well-appearing, immunized children with SFS remains low. Regional variation, age, and clinical presentation will guide the CSF analysis decision. Hence, a selective, case-based approach is preferable for over routine testing. Future studies must focus on refining the risk stratification protocols and validating novel biomarkers for improving diagnostic accuracy and also minimizing unnecessary invasive procedures in pediatric care.

## Appendices

Appendix A

**Table 3:** PRISMA checklist PRISMA: Preferred Reporting Items for Systematic Reviews and Meta-Analyses

Databases searched	PubMed, Cochrane Library, Web of Science, Embase, Google Scholar
Time frame	January 1, 2015-May 2025
Filters applied	Language: English
	Study type: observational studies (cohort, cross-sectional, chart reviews)
	Species: humans
	Age group: 6 to 60 months (infants and young children)
PubMed	
Search string	("febrile seizures"[MeSH Terms] OR "febrile convulsions" OR "fever seizure") AND ("cerebrospinal fluid"[MeSH Terms] OR "CSF analysis" OR "lumbar puncture" OR "spinal tap") AND ("infant"[MeSH Terms] OR "child"[MeSH Terms] OR "pediatric" OR "paediatric")
Filters applied	English; humans; publication date from 2015/01/01 to 2025/05/31
Articles identified	89
Cochrane Library	
Search string	febrile seizures AND CSF analysis AND children
Filtered by	Trials; English language; years 2015-2025
Articles identified	6
Web of Science	
Search string	TS=("febrile seizures" OR "febrile convulsions") AND TS=("CSF" OR "lumbar puncture") AND TS=("child" OR "pediatric")
Refined by	Document type (article); language (English); timespan: 2015-2025
Articles identified	33
Google Scholar	
Search string	All in title: "febrile seizures" AND "cerebrospinal fluid" AND "children"
Filters	Custom date range (2015-2025); sorted by relevance
Articles identified	1137
Embase	
Search string	('febrile seizure'/exp OR 'febrile convulsion' OR 'fever seizure') AND ('cerebrospinal fluid analysis'/exp OR 'lumbar puncture' OR 'spinal tap') AND ('infant'/exp OR 'child'/exp OR 'pediatric patient')
Filters	Human; English; publication years 2015-2025, study types=observational studies
Articles identified	41

Appendix B

**Table 4:** List of excluded studies with reasons CSF: cerebrospinal fluid; CNS: central nervous system; EEG: electroencephalogram

S. no.	Reason for exclusion	Number of studies
1	Age of participants not within 6-60 months	6
2	Included patients with known neurological conditions or CNS infections	5
3	Did not perform or report CSF analysis or lumbar puncture	7
4	Outcomes not related to febrile seizures (e.g., EEG, imaging-focused, epilepsy-only data)	4
5	Incomplete or low-quality data (e.g., abstract-only, inaccessible full text)	6
6	Unsuitable study design (e.g., case report, review, editorial, animal study)	7
